# Vision-Related Quality of Life and Appearance Concerns Are Associated with Anxiety and Depression after Eye Enucleation: A Cross-Sectional Study

**DOI:** 10.1371/journal.pone.0136460

**Published:** 2015-08-28

**Authors:** Juan Ye, Lixia Lou, Kai Jin, Yufeng Xu, Xin Ye, Timothy Moss, Hayley McBain

**Affiliations:** 1 Department of Ophthalmology, Second Affiliated Hospital of Zhejiang University, College of Medicine, Hangzhou, Zhejiang, China; 2 Centre for Appearance Research, Faculty of Health and Life Science, University of the West of England, Bristol, United Kingdom; 3 School of Health Sciences, City University London, London, United Kingdom; School of Ophthalmology and Optometry and Eye Hospital, Wenzhou Medical University, Wenzhou, Zhejiang, CHINA

## Abstract

**Aims:**

To investigate the association of demographic, clinical and psychosocial variables with levels of anxiety and depression in participants wearing an ocular prosthesis after eye enucleation.

**Methods:**

This cross-sectional study included 195 participants with an enucleated eye who were attending an ophthalmic clinic for prosthetic rehabilitation between July and November 2014. Demographic and clinical data, and self-reported feelings of shame, sadness and anger were collected. Participants also completed the National Eye Institute Visual Function Questionnaire, the Facial Appearance subscale of the Negative Physical Self Scale, and the Hospital Anxiety and Depression Scale. Regression models were used to identify the factors associated with anxiety and depression.

**Results:**

The proportion of participants with clinical anxiety was 11.8% and clinical depression 13.8%. More anxiety and depression were associated with poorer vision-related quality of life and greater levels of appearance concerns. Younger age was related to greater levels of anxiety. Less educated participants and those feeling more angry about losing an eye are more prone to experience depression. Clinical variables were unrelated to anxiety or depression.

**Conclusions:**

Anxiety and depression are more prevalent in eye-enucleated patients than the general population, which brings up the issues of psychiatric support in these patients. Psychosocial rather than clinical characteristics were associated with anxiety and depression. Longitudinal studies need to be conducted to further elucidate the direction of causality before interventions to improve mood states are developed.

## Introduction

Eye enucleation may be necessary due to severe ocular trauma, intraocular malignancies, and other serious ocular diseases. A review of clinical causes for 1375 globes enucleated in a large third-referral centre in China showed that trauma was the most common cause (62.5%), followed by tumor (28.5%) and surgically treated or untreated ocular diseases (5.7%) [1]. Eye enucleation and prosthesis wear have been associated with deficits in visual function and alterations in facial appearance. This is unsurprising given that the eyes play an important role in both communication and physical attractiveness [2, 3]. Recent studies have shown that patients with monocular vision experience poorer health-related and vision-related quality of life than those with binocular vision [4–7]. Patients who have been living with an ocular prosthesis for some time report feelings of social anxiety and avoid social situations as a result of their altered appearance, and this has been related to feelings of social acceptance and the negative views they hold about the way they look [8]. However, a longitudinal study indicated that orbital implant insertion and prosthesis wear after enucleation are associated with a significant reduction in anxious and depressive symptoms and an improvement in appearance-related social anxiety and social avoidance [9]. These studies have however been limited by their small sample sizes or have failed to assess the impact of multiple demographic, clinical and psychosocial variables and their association with anxiety and depression in parallel. Therefore, the aim of this study was to investigate levels of anxiety and depression in a sample of eye-enucleated patients living with an ocular prosthesis, and explore the association of demographic variables, clinical variables, vision-related quality of life, appearance concerns, and some feelings about losing an eye with anxiety and depression.

## Methods

### Design

This study was a cross-sectional design, involving opportunity sampling of hospital patients, and employing regression analysis.

### Participants

Patients aged 18 years and above, attending the Department of Ophthalmology at the Second Affiliated Hospital of Zhejiang University, Hangzhou, China, for prosthetic rehabilitation between July and November 2014, were eligible to take part in the study. All patients were inserted with an orbital implant and wearing an custom-made ocular prosthesis after eye enucleation. Reasons for exclusion included cataract, glaucoma or other ocular diseases in the remaining eye that might affect visual function, and severe systemic diseases according to patients’ medical records. Patients who had disfigured face from concomitant facial trauma with eyeball were also excluded. Participants who could not fill out the survey (S1 File) independently were provided with support from the investigators.

### Measures

#### Demographic variables

Data were collected on age, gender, residence, marital status, level of education and monthly income.

#### Clinical variables

Participants were asked which eye was enucleated, the cause of anophthalmos and duration of prosthesis wear. The similarity of the prosthesis to the normal eye (0 = not similar at all, 10 = extremely similar) and comfort of wear (0 = not comfortable at all, 10 = extremely comfortable) were rated by participants using a visual analogue scale.

#### Psychosocial variables

Participants completed two standardized questionnaires measuring vision-specific quality of life and appearance-related concerns, and additional three items asking participants how shameful, sad, and angry they felt about losing an eye using a visual analogue scale (0 = not at all, 10 = extremely).

Vision-specific quality of life was measured using the National Eye Institute Visual Function Questionnaire (NEI VFQ) [10] (S2 File). The NEI VFQ is a vision-specific instrument to assess quality of life, consisting of a General Health subscale, and 11 vision-related subscales including: General Vision, Ocular Pain, Near Activities, Distance Activities, Social Functioning, Mental Health, Role Difficulties, Dependency, Driving, Color Vision, and Peripheral Vision. As it is illegal for people with monocular vision to drive in China, the Driving subscale was omitted. The reliability and validity of this Chinese version of the NEI VFQ has been previously established [11]. A vision-specific composite score is calculated by averaging the vision-related subscale scores. Scores range from 0 to 100 with higher scores indicating better functioning.

Appearance concerns were measured using the Facial Appearance subscale of the Negative Physical Self Scale (NPSS-F) [12] (S2 File). The NPSS is a reliable and valid measure of appearance concerns in the Chinese population, including five dimensions: General Appearance, Facial Appearance, Shortness, Fatness and Thinness. Given the alternations in facial appearance after eye removal, we extracted the Facial Appearance subscale to assess the appearance associated concerns in eye-enucleated participants. Items are rated from 0 (never) to 4 (always) with higher scores representing more negative ratings. The NPSS-F score is calculated by averaging the item scores, with a score of 2 or above implying dissatisfaction with facial appearance [12].

#### Primary outcome measures

Anxiety and depression were assessed using the Hospital Anxiety and Depression Scale (HADS) [13, 14] (S2 File). The HADS is a widely used psychometrically sound scale measuring the anxiety and depression levels in patients with physical health problems. The HADS contains 14 items categorized into two subscales: anxiety and depression (7 items for each scale). Items are scored from 0 (never) to 3 (almost) with higher scores indicating greater levels of anxiety or depression. Interpretation of the subscale scores (range from 0 to 21) was based on the following categories indicating clinical levels of anxiety and depression: 0 to 7, normal; 8 to 10, moderate; and 11 to 21, caseness [13].

### Statistical analyses

Statistical analyses for descriptive statistics were performed using SPSS 20 (IBM, Chicago, IL, USA). Stepwise multiple linear regressions were conducted to identify the unique predictors of levels of anxiety and depression. Statistical power analysis using GPower 3.1.9 indicates a sample size of 175 with α = 0.05 and statistical power (1-β) = 0.9 [15]. The variables outlined in Table 1 were entered into the stepwise regression models. Collinearity statistics, including tolerance and variance inflation factor (VIF) of the independent variables, were used to detect multicollinearity [16]. A tolerance of less than 0.20 or 0.10 and/or a VIF of 5 or 10 and above indicates a multicollinearity problem [17].

**Table 1:** Variables entered into the stepwise regression models.

Demographic variables	Clinical variables	Psychosocial variables	Outcome measures
Age	Enucleated eye	Vision-related QoL	Anxiety
Gender	Etiology	Appearance concerns	Depression
Residence	Duration of wear	Shame	
Marital status	Similarity of prosthesis	Sadness	
Level of education	Comfort of wear	Anger	
Monthly income			

QoL: quality of life.

### Ethical approval

This study was conducted with the approval of the Second Affiliated Hospital of Zhejiang University Institutional Review Board and consistent with the Declaration of Helsinki. All participants provided written informed consent.

## Results

### Descriptive statistics

One hundred and ninety-five (81.9%) of 238 patients consented to take part in the study and complete the cross-sectional survey (S3 File). Table 2 shows the descriptive statistics for demographic, clinical and psychosocial variables, and primary outcome measures. Forty-five participants (23.1%) scored 2 or above on the NPSS-F scale, implying dissatisfaction with their facial appearance. Thirteen (6.7%) participants experienced both clinical anxiety and depression.

**Table 2:** Descriptive statistics for demographic, clinical and psychosocial variables, and primary outcome measures.

Characteristic	N (%)	Range	M (SD)
Age (Year)		18–70	36.3 (12.6)
Gender			
Male	131 (67.2)		
Female	64 (32.8)		
Residence			
Urban area	39 (20.0)		
Rural area	156 (80.0)		
Marital status			
Married	126 (64.6)		
Single, divorced, or widowed	69 (35.4)		
Level of education			
Primary school	25 (12.8)		
Junior high school	80 (41.0)		
Senior high school	38 (19.5)		
University	52 (26.7)		
Monthly income (RMB)			
≤1000	45 (23.1)		
1000–3000	54 (27.7)		
3000–5000	58 (29.7)		
> 5000	38 (19.5)		
Enucleated eye			
Left	94 (48.2)		
Right	101 (51.8)		
Cause of anophthalmos			
Trauma	128 (65.6)		
Eye malignancy	7 (3.6)		
Eye disease	60 (30.8)		
Duration of wear (Month)		2–649	56.3 (96.7)
Similarity of prosthesis		0–10	4.9 (2.4)
Comfort of wear		0–10	5.5 (2.5)
Vision-specific composite		31.5–100.0	70.3 (16.6)
Appearance concern		0–4	1.3 (0.9)
Shame		0–10	4.4 (3.5)
Sadness		0–10	5.1 (3.8)
Anger		0–10	3.7 (3.7)
Anxiety		0–21	6.3 (4.0)
Normal	117 (60.0)		
Moderate	55 (28.2)		
Caseness	23 (11.8)		
Depression		0–16	5.6 (4.0)
Normal	134 (68.7)		
Moderate	34 (17.4)		
Caseness	27 (13.8)		

M: mean; SD: standard deviation.

Stepwise multiple regressions were performed to identify the variables significantly associated with clinical levels of anxiety and depression. Table 3 provides a summary of the statistically significant predictors of anxiety and depression in this population.

**Table 3:** Summary of results from the stepwise regressions analyses.

	Anxiety β (*p*)	Depression β (*p*)
Age	-0.140 (0.013)	-
Level of education	-	-0.221 (<0.001)
Vision-specific composite	-0.506 (<0.001)	-0.346 (<0.001)
Appearance concern	0.359 (<0.001)	0.152 (0.016)
Anger	-	0.217 (0.001)

### Predictors of anxiety

The final regression model explained 50.0% of the variance in anxiety (Adjusted R^2^ = 0.500, F _(3,191)_ = 65.76, *p* <0.001). Standardized β coefficients indicated that age, vision-specific quality of life and appearance concerns each made significant contributions to the final model. Greater anxiety was associated with younger age, poorer vision-related quality of life and greater levels of appearance concerns. The collinearity statistics indicated the absence of multicollinearity, with tolerance ranging from 0.712 to 0.835, and VIF ranging from 1.198 to 1.404.

### Predictors of depression

The final regression model explained 42.7% of the variance in depression (Adjusted R^2^ = 0.427, F _(4,190)_ = 37.10, *p* <0.001). Level of education, vision-specific quality of life, appearance concerns and anger all made significant contributions as indicated by the standardized β coefficients. This suggests that greater levels of depression were associated with lower levels of education, poorer vision-related quality of life, greater levels of appearance concerns and more feelings of anger. Tolerance (ranging from 0.693 to 0.915), and VIF (ranging from 1.093 to 1.443) showed no evidence for multicollinearity between the model variables.

## Discussion

This study aimed to identify the factors associated with mood states in participants living with an ocular prosthesis. Contrary to our expectations, levels of anxiety and depression were not related to gender, cause of anophthalmos, duration and comfort of prosthesis wear, or similarity of prosthesis to the normal eye. Rather greater anxiety and depression were associated with poorer vision-related quality of life and greater concerns about facial appearance. The prevalence rates of anxiety and depression in our sample were 11.8% and 13.8%, respectively, which are significantly higher than the normal levels in the general Chinese population (anxiety: 2.4%, depression: 1.4%) [18], bringing up the issues of psychiatric support in patients who are anophthalmic.

The mean vision-specific composite score of 70.3 in this study was lower than scores of approximately 80 in both Kondo et al’s and Hirneiss et al’s studies, which may be due to distinct participant characteristics and rehabilitation programs [6, 7]. Many aspects of vision-related quality of life are associated with mood states after eye removal. Take ocular pain as an example, in a survey-based study of 112 eye-amputated patients, 26% reported pain symptom, which is a part of phantom eye syndrome [19]. Patients with pain are more prone to a chronic course of anxiety and depression disorders [20]. Also, monocular patients had reported role difficulty and social functioning impairment in their daily life, such as difficulty participating in hobbies, negative feelings regarding social interpersonal relationship, and job performance deficits, which were related to greater anxiety and depression [4, 7, 21].

Greater concern about facial appearance was associated with greater levels of anxiety and depression. Facial features are central to judgments of attractiveness in China, and increasing numbers of Chinese undergo facial cosmetic surgeries to improve their prospects in the workplace and intimate relationships [22, 23]. It is unsurprising that visible disfigurement can result in a range of psychosocial difficulties, especially for those patients with disfiguring eye conditions [24]. At the time of eye enucleation, an implant is usually inserted. Early insertion of ocular prosthesis allows for cosmetic rehabilitation [25], and has been suggested to be associated with improvement in psychological well-being [9, 26]. This study did not collect data on how soon a prosthesis was inserted, but would be an important consideration when interpreting these results. The similarity of prosthesis to the normal eye had insignificant relationship with anxiety and depression, which might be contrary to the general expectation of majority of oculoplastic surgeons who try to achieve this similarity. The correlation analysis indicated that there was no significant correlation between the similarity of the prosthesis to the normal eye and facial appearance concern (r = -0.109, *p* > 0.05). The NPSS-F scale has three sub-dimensions reflecting specific modes of functioning, namely cognition-affect, behavior and projection [12]. Therefore, the assessment of facial appearance concern tends to encompass more than the simple assessment of the similarity of the prosthesis to the normal eye.

Among the demographic factors which we assessed, younger age was associated with greater levels of anxiety. There is some evidence that ageing is related to an intrinsic reduction in susceptibility to anxiety, possibly because of decreased emotional responsiveness with age, increased emotional control and psychological immunization to stressful experience [27]. In addition, lower levels of education were related to greater levels of depression. Some studies of service use have noted that a higher education was positively associated with making mental health visits [28, 29]. Our data suggested that clinical variables alone failed to explain the majority of variance in anxiety and depression. Similar findings have been reported for another disfiguring eye condition such as strabismus [30]. This study also indicated that more anger was associated with being more depressed. Anger has been suggested to be a highly prevalent marker of more severe, chronic, and complex depressive illnesses [31]. Patients living with an ocular prosthesis have reported feelings of social anxiety and avoid social situations [8]. This avoidance can lead to feelings of anger, bitterness, depression, and isolation [32].

This study benefits from a comparatively large sample, with sufficient statistical power. However, the shortcomings of relying on self-reported measures should be noted. Meanwhile, we are aware of the limitations of a cross-sectional study, which limits attributions about the direction of causality between variables. Besides, the findings were restricted to Chinese monocular patients and may not be generalizable to other ethnic groups.

In summary, this study suggested that anxiety and depression are more prevalent in eye-enucleated patients than general population, while it is worth mentioning that the prevalence rates of anxiety and depression vary with age, levels of education, and other patients’ conditions. Psychosocial rather than clinical characteristics are associated with mood states in these patients. Consideration should be given to how psychological health services can be delivered particular for those with poorer quality of life and greater appearance concerns. Longitudinal studies need to be conducted to further elucidate issues of causality before interventions to improve mood states are developed and evaluated.

## Supporting Information

S1 File Chinese survey version. (PDF)

S2 File NEI VFQ, NPSS-F, HADS in English. (DOCX)

S3 File Original data. (XLSX)
