# Supplementary material for: Vision-Related Quality of Life and Appearance Concerns Are Associated with Anxiety and Depression after Eye Enucleation: A Cross-Sectional Study
Source: PLoS One. 2015 Aug 28;10(8):e0136460. doi: 10.1371/journal.pone.0136460 (PMC4552790; doi:10.1371/journal.pone.0136460)
Supplement: S1 File — (PDF) [file pone.0136460.s001.pdf]

知情同意书

尊敬的患者：

我们邀请您参加一项“ 眼球摘除患者的生活状态及心理健康状态 ”的临床研究，在您决定是否参加这项研究之前，请仔细阅读以下内容，它可以帮助您了解该项研究以及为什么要进行这项研究，研究的程序和期限，参加研究后可能给您带来的益处、风险和不便。

以下是本项研究的介绍：

一、研究背景和研究目的

眼球摘除是各种眼病和眼外伤最不幸的结果，该手术改变了病人的视觉功能和容貌，影响患者的正常生活和身心健康。本研究将对单眼眼球摘除患者的生活状态及心理健康状态进行问卷调研，为针对这类患者采取有效的干预措施提供依据。

二、具体程序和流程

本研究将采集您的相关病史资料，并需要您填写一份关于生活状态和心理健康状态的调查问卷，我们将对每位受试者进行编号，建立病历档案。

三、如果参加研究您需要做什么

您需要提供有关自身病史和当前生活、心理状态的真实情况；告诉研究者在本次研究期间所出现的任何不适；告诉研究者最近是否曾参与其他研究，或目前正参与其他研究。

四、参加本研究可能给您带来的受益

您参与本研究具有重要的社会意义，研究结果将有利于我们更深入地了解眼球摘除患者的生活状态和心理健康状态，以便为眼球摘除患者提供更多的社会心理支持。

五、参加本研究可能发生的不良反应、风险以及风险防范措施

本研究需要被试者填写一份调查问卷，不会带来额外的风险。如有不良事件发生，及时呈报上级主管部门。

六、费用情况说明

任何因研究为目的而进行的程序，您无需支付其他额外费用。

七、参与研究的补偿，包括损伤的赔偿

患者参加本研究无相关经济补偿；若发生与本研究内容相关损伤能获得相应赔偿。

八、替代方案

如果不参加本研究，不存在替代方案，不会影响您的常规临床治疗。

九、您个人信息的保密

您的医疗记录（包括研究病历及理化检查报告等）将按规定保存在医院。除研究者、伦理委员会、监查、稽查、药政管理部门等相关人员将被允许查阅您的医疗记录外，其他与研究无关的人员在未得到允许的情况下，无权查阅您的医疗记录。本研究结果的公开报告将不会披露您的个人身份。我们将在允许的范围内，尽一切努力保护您个人医疗资料的隐私。

十、终止参加研究

是否参加本项研究完全取决于您的自愿。您可以拒绝参加此项研究，或在研究过程中的任何时间无理由退出研究，这都不会影响您和医生的关系，都不会影响对您的医疗或有其他方面利益的损失。此外，由于以下原因，可能会终止您参与本研究：

1、您未遵从研究医生的医嘱。

2、您发生了可能需要治疗的严重情况。

3、研究医生认为，终止研究对您的健康和福利最有利。

十一、伦理委员会

本研究已向浙江大学医学院附属第二医院人体研究伦理委员会报告，经委员会的全面审查和包括对受试者的风险评估，并获得了批准。在研究中过程中，**有关伦理和权益事宜可联系浙江大学医学院附属第二医院人体研究伦理委员会，电话：白天 0571-87783759； 晚上（总值班）：13757118366；邮箱地址：** HREC2013@126.com

---

我确认已阅读并理解了本研究的知情同意书，自愿接受本研究中的治疗方法，并同意将我的医疗数据用于本研究的发表。

受试者签名：__________ 联系方式：______________ 日期：_____________

代理人签名：______________与受试者关系______________联系方式_____________ 日期______________

（如果需要）

见证人（如果需要）：_____________联系方式：______________ 日期：_____________

我确认已向患者解释了本研究的详细情况，包括其权利以及可能的受益和风险，并给其一份签署过的知情同意书副本。

研究者签名：_____________

联系方式：______________（手机） 日期：________________

您的个人资料

☆请正确填写个人资料，并在合适的选项☐内打 √

姓名：	就诊号：
性别：☐男 ☐女	年龄：
来自：☐城镇 ☐农村	职业：
婚姻：☐未婚 ☐已婚 ☐离异 ☐丧偶	手机号码：
学历：☐小学及以下 ☐初中 ☐高中/中专 ☐本科/大专 ☐硕士及以上	月收入：☐1000 元及以下 ☐1001-3000 元 ☐3001-5000 元 ☐5000 元以上
摘除的眼球：☐左眼 ☐右眼	眼球摘除的日期（年/月）：
佩戴义眼：☐是 ☐否	第一次佩戴义眼的日期（年/月）：
摘除的原因：☐眼外伤 ☐眼肿瘤 ☐眼球失明伴疼痛 ☐眼球失明有碍美观	

以下每个问题的下方有一个 **0 至 10 十个刻度的标尺**。请仔细阅读题目，并在标尺上圈出一个数字，表示您目前的真实情况。

1. 因为眼球摘除的原因，**您的工作**受到了多大程度的影响？
（0 表示毫无影响，10 表示巨大影响，请在 0 至 10 之间圈出一个数字）

毫无影响 巨大影响
0 1 2 3 4 5 6 7 8 9 10

2. 因为眼球摘除的原因，**您的收入**是否减少？（圈出一个数字）
（0 表示毫无减少，10 表示大幅减少，请在 0 至 10 之间圈出一个数字）

毫无减少 大幅减少
0 1 2 3 4 5 6 7 8 9 10

3. 因为眼球摘除的原因，**您和亲人的关系**受到了多大程度的影响？（圈出一个数字）

毫无影响 巨大影响
0 1 2 3 4 5 6 7 8 9 10

4. 因为眼球摘除的原因，**您和朋友的关系**受到了多大程度的影响？（圈出一个数字）

毫无影响 巨大影响
0 1 2 3 4 5 6 7 8 9 10

5. 目前您摘除的眼球是否有**不适感**（例如灼热、痒或疼痛）？（圈出一个数字）

毫无不适 |___|___|___|___|___|___|___|___|___|___| 非常不适
0 1 2 3 4 5 6 7 8 9 10

6. 因为眼球摘除的原因，您是否感到**羞愧**？（圈出一个数字）

毫无羞愧 |___|___|___|___|___|___|___|___|___|___| 非常羞愧
0 1 2 3 4 5 6 7 8 9 10

7. 因为眼球摘除的原因，您是否感到**悲伤**？（圈出一个数字）

毫无悲伤 |___|___|___|___|___|___|___|___|___|___| 非常悲伤
0 1 2 3 4 5 6 7 8 9 10

8. 因为眼球摘除的原因，您是否感到**愤怒**？（圈出一个数字）

毫无愤怒 |___|___|___|___|___|___|___|___|___|___| 非常愤怒
0 1 2 3 4 5 6 7 8 9 10

9. 因为眼球摘除的原因，您是否产生了**心理压力**？（圈出一个数字）

毫无压力 |___|___|___|___|___|___|___|___|___|___| 巨大压力
0 1 2 3 4 5 6 7 8 9 10

~~~~~~~~~~~~~~~~~~~~请佩戴过义眼的患者，回答以下问题~~~~~~~~~~~~~~~~~~~~

10. 您感觉目前佩戴义眼的**舒适度**为：（圈出一个数字）

毫不舒适 |___|___|___|___|___|___|___|___|___|___| 非常舒适
0 1 2 3 4 5 6 7 8 9 10

11. **从外观上看**，您感觉义眼与正常眼的**相似度**为： （圈出一个数字）

毫不相似 |___|___|___|___|___|___|___|___|___|___| 非常相似
0 1 2 3 4 5 6 7 8 9 10

12. 总的来说，您对目前佩戴的义眼**满意度**为： （圈出一个数字）

毫不满意 |___|___|___|___|___|___|___|___|___|___| 非常满意
0 1 2 3 4 5 6 7 8 9 10

视觉功能量表

以下一些句子的内容涉及与您视力有关的问题或您对自己视力情况的感受。在每条问题后，会有一连串可能的答案。请选择最能形容您的情况之答案。

请想象自己正在佩戴眼镜或隐形眼镜（如有的话）的情况下来回答所有问题。您需要用多少时间来回答每条问题都可以。

您所有的答案将会保密。您的答案必须尽量准确，我们才能透过这项调查来增进我们对视力问题的认识及它如何影响您的生活质量。请记着：**如果您在进行某项活动时需要佩戴眼镜或隐形眼镜，请根据已佩戴它们的情况回答以下问题**。

第一部份 – 整体健康及视力

1. 一般来说，您的整体健康属于：（圈出一个答案）

极佳……………………………1
非常好…………………………2
良好……………………………3
一般……………………………4
差………………………………5

2. 现在来说，仅用单眼（如您需佩戴眼镜或隐形眼镜，则根据已佩戴的情况作答）您认为自己的视力属于：（圈出一个答案）

极佳……………………………1
良好……………………………2
一般……………………………3
差………………………………4
非常差…………………………5
完全失明………………………6

3. 您有多少时间会为自己的视力担忧？（圈出一个答案）

从来没有………………………1
少许时间………………………2
有时……………………………3
大部分时间……………………4
全部时间………………………5

4. 您感到眼睛及它周围有多大的痛楚或不适（例如灼热、痒或疼痛）？（圈出一个答案）

完全没有………………………1
轻微……………………………2
中度……………………………3
严重……………………………4
非常严重………………………5

第二部份 – 活动困难

以下的问题是关于您在进行某些活动时遇到多大的困难（如您需佩戴眼镜或隐形眼镜，则根据已佩戴的情况作答）

根据下列句子适合你自己的程度，在右边的选项中“圈出相应的数字”。

完全没有困难	有少许困难	有中度困难	有极大困难	因视力的原故已停止此活动	因其他理由或对所述活动没有兴趣，已停止此活动
1	2	3	4	5	6

5. 您在阅读报章上一般的字体时有多大困难？	1	2	3	4	5	6
6. 您进行在近距离需要看得清楚的工作或嗜好时（例如下厨、缝纫、家居修补或使用手工具）有多大困难？	1	2	3	4	5	6
7. 因视力的原故，您在放满对象的架上寻找东西时有多大困难？	1	2	3	4	5	6
8. 您在阅读路牌或店铺名称时有多大困难？	1	2	3	4	5	6
9. 因视力的原故，您在灯光阴暗的环境、或晚上下台阶、梯级或行人路边有多大困难？	1	2	3	4	5	6
10. 因视力的原故，您一路走时察觉到两旁的物件有多大困难？	1	2	3	4	5	6
11. 因视力的原故，您要观察到别人对您所说的话的反应有多大困难？	1	2	3	4	5	6
12. 因视力的原故，您在挑选及配衬自己的衣服时有多大困难？	1	2	3	4	5	6
13. 因视力的原故，您到别人家中探望他们、在派对或餐厅里与人共聚有多大困难？	1	2	3	4	5	6
14. 因视力的原故，您在观看及欣赏电视节目时有多大困难？	1	2	3	4	5	6

第三部分：对视力问题的响应

以下的问题是关于您的视力对您所做的事可能产生的影响。就每条问题，请告诉我它对您来说是：任何时间都正确、大部分时间正确、有时正确、少许时间正确或从来都不正确。

根据下列句子适合你自己的程度，在右边的选项中“圈出相应的数字”。

任何时间	大部分时间	有时	少许时间	从不
1	2	3	4	5

17. 因视力的原故，您能完成的事情是否比您想做的少？	1	2	3	4	5
18. 因视力的原故，您工作或进行其他活动多长时间会受到限制？	1	2	3	4	5
19. 您的眼睛及它周围的痛楚或不适（例如灼热、痒或疼痛）在多大程度上令您不能做您想做的事？	1	2	3	4	5

就以下每句，请告诉我它对您来说是：肯定对、大部分对、不肯定、大部分不对、或肯定不对。

根据下列句子适合你自己的程度，在右边的选项中“圈出相应的数字”。

肯定对	大部分对	不肯定	大部分不对	肯定不对
1	2	3	4	5

20. 因视力的原故，我大部分时间都留在家中。	1	2	3	4	5
21. 因视力的原故，我很多时间感到沮丧。	1	2	3	4	5
22. 因视力的原故，我对自己所做的事情的控制能力少了很多。	1	2	3	4	5
23. 因视力的原故，我需要过度依赖听别人所告诉我的话。	1	2	3	4	5
24. 因视力的原故，我需要别人给予我许多的帮助。	1	2	3	4	5
25. 因视力的原故，我担心会做出一些令自己或别人尴尬的事。	1	2	3	4	5

副量表：整体健康

A1. 在一个以0代表情况与死亡一样差，而10代表可能有的最佳健康状况的量表上，您会给您的整体健康多少评分？（圈出一个答案）

最差 0 1 2 3 4 5 6 7 8 9 10 最佳

副量表：整体视力

A2. 在一个以0代表最差的视力，即无异于失明甚或更坏，而10代表视力处于最佳状态的量表上，您会给您现在的视力多少评分(如您需佩戴眼镜或隐形眼镜，则根据已佩戴的情况作答)？（圈出一个答案）

最差 0 1 2 3 4 5 6 7 8 9 10 最佳

医院焦虑抑郁量表

情绪在大多数疾病中起着重要作用，如果医生了解您的情绪变化，他们就能给您更多的帮助。请您阅读以下各个项目，并根据自己在**过去一段时间内的感受**在相应选项□内**打√**。对这些问题的回答不要做过多的考虑，立即做出的回答往往更符合实际情况。

1.我感到紧张（或痛苦）。	□ 几乎所有时候
	□ 大多数时候
	□ 有时
	□ 根本没有
2.我对以往感兴趣的事情还是有兴趣。	□ 肯定一样
	□ 不像以前那样多
	□ 只有一点儿
	□ 基本上没有了
3.我感到有点害怕，好像预感到什么可怕事情要发生。	□ 非常肯定和十分严重
	□ 是有，但并不太严重
	□ 有一点，但并不使我苦恼
	□ 根本没有
4.我能够哈哈大笑，并看到事物好的一面。	□ 我经常这样
	□ 现在已经不大这样了
	□ 现在肯定是不太多了
	□ 根本没有
5.我的心中充满烦恼。	□ 大多数时间
	□ 常常如此
	□ 时时，但并不经常
	□ 偶然如此
6.我感到愉快。	□ 根本没有
	□ 并不经常
	□ 有时
	□ 大多数
7.我能够安闲而轻松地坐着。	□ 肯定
	□ 经常
	□ 并不经常
	□ 根本没有

8. 我对自己的仪容（打扮自己）失去兴趣。	☐ 肯定不关心
	☐ 并不像我应该做的那样关心
	☐ 我可能不是非常关心
	☐ 我仍像以前一样关心
9. 我有点坐立不安，好像感到非要活动不可。	☐ 确实非常多
	☐ 是不少
	☐ 并不很多
	☐ 根本没有
10. 我对一切都是乐观地向前看。	☐ 差不多是这样做
	☐ 并不完全是这样做的
	☐ 很少这样做
	☐ 几乎从不这样做
11. 我突然发现恐慌感。	☐ 确实很经常
	☐ 时常
	☐ 并非经常
	☐ 根本没有
12. 我好像感到情绪在渐渐低落。	☐ 几乎所有时间
	☐ 很经常
	☐ 有时
	☐ 根本没有
13. 我感到有点害怕，好像某个内脏器官变坏了。	☐ 非常经常
	☐ 很经常
	☐ 有时
	☐ 根本没有
14. 我能欣赏一本好书或意向好的广播或电视节目。	☐ 常常如此
	☐ 有时
	☐ 并非经常
	☐ 很少

负面身体自我量表

问卷中的每个题目是从不同的侧面，对自己长相的种种评价。由于每个人对自己的认识和感受的不同，因而对每个问题的回答也不同。请你仔细阅读问卷，注意：（1）每道题都要回答，不要有遗漏，也不必费时去考虑；（2）要根据你的实际情况回答。

注：题目中的“长相”专指脸，不包括身材。

根据下列句子适合你自己的程度，在右边的选项中“圈出相应的数字”。

从不	少有	有时	常常	总是
1	2	3	4	5

1. 我担心自己的长相。	1	2	3	4	5
2. 如果有什么方法可以改变我的长相，我一定能坚持做下去。	1	2	3	4	5
3. 我觉得我最喜欢的人不喜欢我的样子。	1	2	3	4	5
4. 我的长相让我沮丧。	1	2	3	4	5
5. 我对自己的长相感到羞愧。	1	2	3	4	5
6. 我的同龄人不喜欢我的样子。	1	2	3	4	5
7. 我能感觉到别人对我的长相不满意。	1	2	3	4	5
8. 如果有可能，我会改变我的长相的许多方面。	1	2	3	4	5
9. 如果条件许可，我会作美容手术。	1	2	3	4	5
10. 我周围的人都不喜欢我的长相。	1	2	3	4	5
11. 我不喜欢镜子中看到的自己。	1	2	3	4	5
