# Supplementary material for: Vision-Related Quality of Life and Appearance Concerns Are Associated with Anxiety and Depression after Eye Enucleation: A Cross-Sectional Study
Source: PLoS One. 2015 Aug 28;10(8):e0136460. doi: 10.1371/journal.pone.0136460 (PMC4552790; doi:10.1371/journal.pone.0136460)
Supplement: S2 File — (DOCX) [file pone.0136460.s002.docx]

**The National Eye Institute Visual Function Questionnaire**

The following is a survey with statements about problems which involve your vision or feelings that you have about your vision condition. After each question please choose the response that best describes your situation.

Please answer all the questions as if you were wearing your glasses or contact lenses (if any).

Please take as much time as you need to answer each question. All your answers are confidential. In order for this survey to improve our knowledge about vision problems and how they affect your quality of life, your answers must be as accurate as possible. Remember, if you wear glasses or contact lenses, please answer all of the following questions as though you were wearing them.

INSTRUCTIONS:

1. In general we would like to have people try to complete these forms on their own. If you find that you need assistance, please feel free to ask the project staff and they will assist you.

2. Please answer every question.

3. Answer the questions by circling the appropriate number.

4. If you are unsure of how to answer a question, please give the best answer you can and make a comment in the left margin.

5. Please complete the questionnaire before leaving the center and give it to a member of the project staff. Do not take it home.

6. If you have any questions, please feel free to ask a member of the project staff, and they will be glad to help you.

STATEMENT OF CONFIDENTIALITY:

All information that would permit identification of any person who completed this questionnaire will be regarded as strictly confidential. Such information will be used only for the purposes of this study and will not be disclosed or released for any other purposes without prior consent, except as required by law.

PART 1 - GENERAL HEALTH AND VISION

1. In general, would you say your overall health is: (Circle One)

Excellent...…………………………1

Very good..…………………………2

Good.………………………………3

Fair…………………………………4

Poor...………………………………5

2. At the present time, would you say your eyesight using the only remaining eye (with glasses or contact lenses, if you wear them) is: (Circle One)

Excellent.……………………………1

Good...………………………………2

Fair..…………………………………3

Poor.…………………………………4

Very poor.……………………………5

Completely blind.……………………6

3. How much of the time do you worry about your eyesight? (Circle One)

None of the time.……………………1

A little of the time...…………………2

Some of the time.……………………3

Most of the time..……………………4

All of the time.………………………5

4. How much pain or discomfort have you had in and around your eyes (for example, burning,

itching, or aching)? (Circle One)

None…………………………………1

Mild.…………………………………2

Moderate .……………………………3

Severe..………………………………4

Very severe..…………………………5

PART 2 - DIFFICULTY WITH ACTIVITIES

The next questions are about how much difficulty, if any, you have doing certain activities wearing your glasses or contact lenses if you use them for that activity. For each one, please circle the number to indicate whether you have no, a little, moderate, extreme difficulty doing certain activities, stopped doing this because of your eyesight, or stopped doing this for other reasons or not interested in doing this.

| No  difficulty  at all | A little difficulty | Moderate difficulty | Extreme difficulty | Stopped doing this because of your eyesight | Stopped doing this for other reasons or not interested in doing this |
| --- | --- | --- | --- | --- | --- |
| 1 | 2 | 3 | 4 | 5 | 6 |

| 5. How much difficulty do you have reading ordinary print in  newspapers? | 1 2 3 4 5 6 |
| --- | --- |
| 6. How much difficulty do you have doing work or hobbies that require  you to see well up close, such as cooking, sewing, fixing things  around the house, or using hand tools? | 1 2 3 4 5 6 |
| 7. Because of your eyesight, how much difficulty do you have finding  something on a crowded shelf? | 1 2 3 4 5 6 |
| 8. How much difficulty do you have reading street signs or the names of  stores? | 1 2 3 4 5 6 |
| 9. Because of your eyesight, how much difficulty do you have going  down steps, stairs, or curbs in dim light or at night? | 1 2 3 4 5 6 |
| 10. Because of your eyesight, how much difficulty do you have noticing  objects off to the side while you are walking along? | 1 2 3 4 5 6 |
| 11. Because of your eyesight, how much difficulty do you have seeing  how people react to things you say? | 1 2 3 4 5 6 |
| 12. Because of your eyesight, how much difficulty do you have picking  out and matching your own clothes? | 1 2 3 4 5 6 |
| 13. Because of your eyesight, how much difficulty do you have visiting  with people in their homes, at parties, or in restaurants? | 1 2 3 4 5 6 |
| 14. Because of your eyesight, how much difficulty do you have  watching television? | 1 2 3 4 5 6 |

PART 3: RESPONSES TO VISION PROBLEMS

The next questions are about how things you do may be affected by your vision. For each one, please circle the number to indicate whether the statement is true for you all, most, some, a little, or none of the time.

| All of the time | Most of the time | Some of the time | A little of the time | None of the time |
| --- | --- | --- | --- | --- |
| 1 | 2 | 3 | 4 | 5 |

| 17. Do you accomplish less than you would like because of your vision? | 1 2 3 4 5 |
| --- | --- |
| 18. Are you limited in how long you can work or do other activities  because of your vision? | 1 2 3 4 5 |
| 19. How much does pain or discomfort in or around your eyes, for example, burning, itching, or aching, keep you from doing what you’d like to be doing? | 1 2 3 4 5 |

For each of the following statements, please circle the number to indicate whether the statement is definitely true, mostly true, mostly false, or definitely false for you or you are not sure.

| Definitely true | Mostly true | Not sure | Mostly false | Definitely false |
| --- | --- | --- | --- | --- |
| 1 | 2 | 3 | 4 | 5 |

| 20. I stay home most of the time because of my eyesight. | 1 2 3 4 5 |
| --- | --- |
| 21. I feel frustrated a lot of the time because of my eyesight. | 1 2 3 4 5 |
| 22. I have much less control over what I do, because of my eyesight. | 1 2 3 4 5 |
| 23. Because of my eyesight, I have to rely too much on what other people tell me. | 1 2 3 4 5 |
| 24. I need a lot of help from others because of my eyesight. | 1 2 3 4 5 |
| 25. I worry about doing things that will embarrass myself or others, because of my eyesight. | 1 2 3 4 5 |

SUBSCALE: GENERAL HEALTH

A1. How would you rate your overall health, on a scale where zero is as bad as death and 10 is best possible health? (Circle One)

0 1 2 3 4 5 6 7 8 9 10

Worst Best

SUBSCALE: GENERAL VISION

A2. How would you rate your eyesight now (with glasses or contact lens on, if you wear them), on a scale of from 0 to 10, where zero means the worst possible eyesight, as bad or worse than being blind, and 10 means the best possible eyesight? (Circle One)

0 1 2 3 4 5 6 7 8 9 10

Worst Best

**The Facial Appearance Subscale of the** **Negative Physical Self Scale**

Instructions: This questionnaire is about how you evaluate your facial appearance from different aspects. For each sentence, please circle the number to indicate whether the statement is never, seldom, sometimes, often, or always true for you. You do not have to think too much to answer. In this questionnaire, spontaneous answers are more important. Mark only one answer for each question.

| Never | Seldom | Sometimes | Often | Always |
| --- | --- | --- | --- | --- |
| 1 | 2 | 3 | 4 | 5 |

| 1. I am worried about my facial appearance. | 1 2 3 4 5 |
| --- | --- |
| 1. If there is some way I can improve my face, I will keep trying to do it. | 1 2 3 4 5 |
| 1. The people I like most do not like the way my face looks. | 1 2 3 4 5 |
| 1. I am depressed about how my face looks. | 1 2 3 4 5 |
| 1. I am ashamed about my facial appearance. | 1 2 3 4 5 |
| 1. My peer group does not like my looks. | 1 2 3 4 5 |
| 1. Others are not satisfied with the way my face appears. | 1 2 3 4 5 |
| 1. If it is possible, I will change the way my face looks. | 1 2 3 4 5 |
| 1. If possible, I will have cosmetic surgery. | 1 2 3 4 5 |
| 1. People around me do not like the way my face looks. | 1 2 3 4 5 |
| 1. I do not like what I see when I look in the mirror. | 1 2 3 4 5 |

**The Hospital Anxiety and Depression Scale**

Instructions: This questionnaire will help your physician know how you are feeling. Read each sentence. Place an “√” on the answer that best describes how you have been feeling during the LAST WEEK. You do not have to think too much to answer. In this questionnaire, spontaneous answers are more important. Mark only one answer for each question.

| 1. I feel tense or wound up. | - Most of the time |
| --- | --- |
|  | - A lot of the time |
|  | - From time to time |
|  | - Not at all |
| 2. I still enjoy the things I used to enjoy. | - Definitely as much |
|  | - Not quite so much |
|  | - Only a little |
|  | - Hardly at all |
| 3. I get a sort of frightened feeling as if something bad is about to happen. | - Very definitely and quite badly |
|  | - Yes, but not too badly |
|  | - A little, but it doesn’t worry me |
|  | - Not at all |
| 4. I can laugh and see the funny side of things. | - As much as I always could |
|  | - Not quite as much now |
|  | - Definitely not so much now |
|  | - Not at all |
| 5. Worrying thoughts go through my mind. | - Most of the time |
|  | - A lot of the time |
|  | - From time to time |
|  | - Only occasionally |
| 6. I feel cheerful. | - Not at all |
|  | - Not often |
|  | - Sometimes |
|  | - Most of the time |
| 7. I can sit at ease and feel relaxed. | - Definitely |
|  | - Usually |
|  | - Not often |
|  | - Not at all |
| 8. I feel as if I am slowed down. | - Nearly all the time |
|  | - Very often |
|  | - Sometimes |
|  | - Not at all |
| 9. **I get a sort of frightened feeling like butterflies in the stomach.** | - Not at all |
|  | - From time to time |
|  | - Quite Often |
|  | - Very Often |
| 10. **I have lost interest in my appearance.** | - Definitely |
|  | - I don't take as much care as I should |
|  | - I may not take quite as much care |
|  | - I take just as much care as ever |
| 11. **I feel restless as I have to be on the move.** | - Very much indeed |
|  | - Quite a lot |
|  | - Not very much |
|  | - Not at all |
| 12. **I look forward with enjoyment to things.** | - As much as I ever did |
|  | - A little less than I used to |
|  | - Definitely less than I used to |
|  | - Hardly at all |
| 13. **I get sudden feelings of panic.** | - Very often indeed |
|  | - Quite often |
|  | - From time to time |
|  | - Not at all |
| 14. I can enjoy a good book or radio or TV programme. | - Often |
|  | - Sometimes |
|  | - Not often |
|  | - Very seldom |
